# High quality draft genome sequence of *Brachymonas chironomi* AIMA4^T^ (DSM 19884^T^) isolated from a *Chironomus* sp. egg mass

**DOI:** 10.1186/s40793-015-0010-4

**Published:** 2015-05-27

**Authors:** Sivan Laviad, Alla Lapidus, James Han, Matthew Haynes, TBK Reddy, Marcel Huntemann, Amrita Pati, Natalia N Ivanova, Konstantinos Mavromatis, Elke Lang, Manfred Rohde, Victor Markowitz, Tanja Woyke, Hans-Peter Klenk, Nikos C Kyrpides, Malka Halpern

**Affiliations:** 1 Dept. of Evolutionary and Environmental Biology, Faculty of Natural Sciences, University of Haifa, Haifa, Israel; 2 Theodosius Dobzhansky Center for Genome Bionformatics, St. Petersburg State University, St. Petersburg, Russia; 3 Algorithmic Biology Lab, St. Petersburg Academic University, St. Petersburg, Russia; 4 DOE Joint Genome Institute, Walnut Creek, CA, USA; 5 Leibniz-Institute DSMZ - German Collection of Microorganisms and Cell Cultures, Braunschweig, Germany; 6 Helmholz Centre for Infection Research, Braunschweig, Germany; 7 Biological Data Management and Technology Center, Lawrence Berkeley National Laboratory, Berkeley, CA, USA; 8 Dept. of Biological Sciences, Faculty of Science, King Abdulaziz University, Jeddah, Saudi Arabia; 9 Dept. of Biology and Environment, Faculty of Natural Sciences, University of Haifa, Oranim, Kiryat Tivon, Israel

**Keywords:** Brachymonas chironomi, Comamonadaceae, Chironomid, Chironomus, Egg mass, Toxicant

## Abstract

*Brachymonas chironomi* strain AIMA4^T^ (Halpern et al., 2009) is a Gram-negative, non-motile, aerobic, chemoorganotroph bacterium. *B. chironomi* is a member of the *Comamonadaceae*, a family within the class *Betaproteobacteria*. This species was isolated from a chironomid (*Diptera; Chironomidae*) egg mass, sampled from a waste stabilization pond in northern Israel. Phylogenetic analysis based on the 16S rRNA gene sequences placed strain AIMA4^T^ in the genus *Brachymonas.* Here we describe the features of this organism, together with the complete genome sequence and annotation. The DNA GC content is 63.5%. The chromosome length is 2,509,395 bp. It encodes 2,382 proteins and 68 RNA genes. *Brachymonas chironomi* genome is part of the *Genomic Encyclopedia of Type Strains, Phase I: the one thousand microbial genomes (KMG) project*.

## Introduction

Strain AIMA4^T^ (= LGM 24400T = DSM 19884T), is the type strain of *Brachymonas chironomi*, one out of two species in the genus *Brachymonas*. The genus *Brachymonas* was formed by Hiraishi et al. [1] while characterizing rhodoquinone-containing bacteria that had been isolated from soybean crude waste sludge in Japan. Strain AIMA4^T^, was isolated from an insect egg mass (*Chironomus* sp*.*) that was sampled from a waste stabilization pond in northern Israel [2]. Chironomids (*Arthropoda*; *Insecta*; *Diptera*; *Chironomidae*; *Chironomus* sp*.*) inhabit virtually every type and condition of aquatic habitats. They undergo a complete metamorphosis of four life stages (egg, larva, pupa and adult that emerges into the air) [3]. Eggs are laid in an egg mass at the water’s edge. Each egg mass contains hundreds of eggs. Chironomid egg masses were found to harbor *Vibrio cholerae* and *Aeromonas* spp. [3]-[10]. *V. cholerae* degrades chironomid egg masses by the secreted haemagglutinin protease (HAP) [11],[12]. Strain AIMA4^T^ was isolated in the course of a study that investigated endogenous bacterial communities that inhabit chironomid egg masses [2],[13],[14]. The species epithet *chironomi* was derived from the non-biting midge insect *Chironomus* (*Diptera; Chironomidae*), from where this species was isolated. Strain AIMA4^T^ didn’t show the ability to degrade the egg masses like it was found for *V. cholerae*.

Here we describe a summary classification and a set of the features of *Brachymonas chironomi* strain AIMA4^T^ (DSM 19884T), together with the genome sequence description and annotation.

## Organism information

### Classification and features

**Figure 1:**
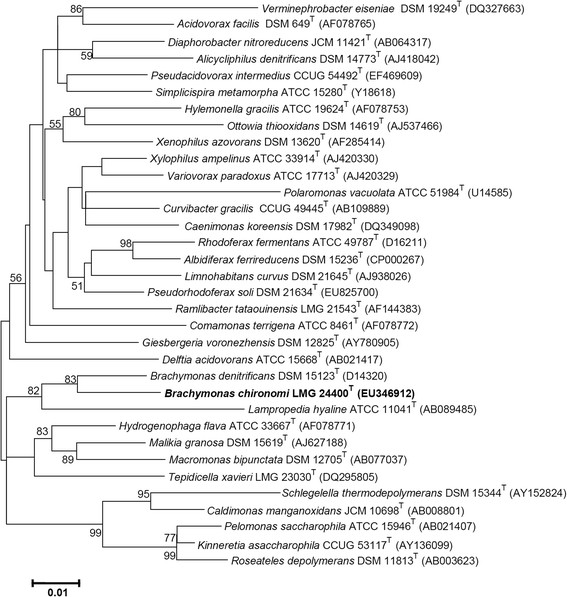
Phylogenetic tree highlighting the position of *Brachymonas chironomi* relative to the type strains of the other species within the family *Comamonadaceae.* The sequence alignments were performed by using the CLUSTAL W program and the tree was generated using the maximum likelihood method in MEGA 5 software. Bootstrap values (from 1,000 replicates) greater than 50% are shown at the branch points. The bar indicates a 1% sequence divergence.

A taxonomic study using a polyphasic approach placed *B. chironomi* strain AIMA4^T^ in the genus *Brachymonas* within the family *Comamonadaceae* (Figure 1). The family *Comamonadaceae* comprises a larger number of genera (as shown in Figure 1) and a larger variety of species and phenotypes [15],[16].

**Figure 2:**
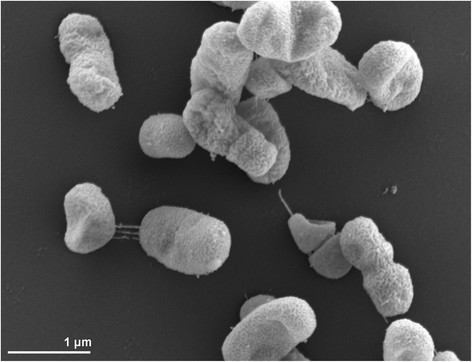
Scanning electron micrograph of *B.chironomi* AIMA4^T^.

*B. chironomi* strain AIMA4^T^ is a Gram-negative, non-motile coccobacillus or rod (Figure 2). After 48 h incubation on LB agar at 30°C, colonies are beige colored (opaque) that turn to light brown after few days of incubation. Strain AIMA4^T^ is aerobic, chemoorganotrophic and does not produce acid from carbohydrates (including glucose) [2]. Growth is observed at 18–37°C (optimum 30°C), with 0–2.5% (w/v) NaCl (optimum 0.5% NaCl) and at pH 5.0–9.0 (optimum pH 6.0–8.0) (Table 1). The following enzymatic activities were observed in strain AIMA4^T^: catalase and oxidase, alkaline and acid phosphatases, esterase (C4), esterase lipase (C8), leucine arylamidase, valine arylamidase, trypsin and naphthol-AS-BI-phosphohydrolase. Strain AIMA4^T^ produces acetoin and reduces nitrate to nitrite [2].

**Table 1:** **Classification and general features of***Brachymonas chironomi* strain AIMA4^T^**according to the MIGS recommendations [**[40]**],** published by the Genome Standards Consortium **[**[41]**] and the Names for Life database [**[42]**]**

**MIGS ID**	**Property**	**Term**	**Evidence code**^**a**^
	Classification	Domain *Bacteria*	TAS [43]
		Phylum *Proteobacteria*	TAS [44]
		Class *Betaproteobacteria*	TAS [45]
		Order *Burkholderiales*	TAS [46]
		Family *Comamonadaceae*	TAS [47]
		Genus *Brachymonas*	TAS [1]
		Species *Brachymonas chironomi*	TAS [2]
		Type strain AIMA4^T^	TAS [2]
	Gram stain	Negative	TAS [2]
	Cell shape	Coccobacilli or rods	TAS [2]
	Motility	Non-motile	TAS [2]
	Sporulation	Non-sporulating	IDS
	Temperature range	18-37°C	TAS [2]
	Optimum temperature	30°C	TAS [2]
	pH range; Optimum	5.0–9.0; 6.0–8.0	TAS [2]
	Carbon source^b^	phenylacetic acid	TAS [2]
MIGS-6	Habitat	Aquatic/Insect host	TAS [2]
MIGS-6.3	Salinity	0-2.5% NaCl (w/v)	TAS [2]
MIGS-22	Oxygen requirement	Aerobic	TAS [2]
MIGS-15	Biotic relationship	Commensal (Insect, chironomid)	TAS [2]
MIGS-14	Pathogenicity	Non-pathogen	NAS
MIGS-4	Geographic location	Israel	TAS [2]
MIGS-5	Sample collection	July, 2006	TAS [2]
MIGS-4.1	Latitude	32.669167	TAS [2]
MIGS-4.2	Longitude	35.128639	TAS [2]
MIGS-4.4	Altitude	40 m	TAS [2]

^a^Evidence codes - IDA: Inferred from Direct Assay; TAS: Traceable Author Statement (i.e., a direct report exists in the literature); NAS: Non-traceable Author Statement (i.e., not directly observed for the living, isolated sample, but based on a generally accepted property for the species, or anecdotal evidence). Evidence codes are from the Gene Ontology project [48].

^b^The only carbon source that was positive for this strain, out of all carbon sources that were tested (strain AIMA4^T^ does not use carbohydrates, not even glucose) [2].

### Chemotaxonomic data

The dominant cellular fatty acids are C_16:1_*ω*7*c*, C_16:0_ and C_18:1_*ω*7*c*. The main isoprenoid quinone is Q-8. Phosphatidylglycerol, phosphatidylethanolamine and phosphatidylserine occur as polar lipids [2].

## Genome sequencing and annotation

### Genome project history

This organism was selected for sequencing on the basis of its phylogenetic position [17]-[19]. Sequencing of *B. chironomi* strain AIMA4^T^ is part of Genomic Encyclopedia of Type Strains, Phase I: the one thousand microbial genomes project [20] which aims in increasing the sequencing coverage of key reference microbial genomes [21]. The genome project is deposited in the Genomes OnLine Database [22] and the permanent draft genome sequence is deposited in GenBank. Sequencing, finishing and annotation were performed by the DOE Joint Genome Institute (JGI) using state of the art sequencing technology [23]. A summary of the project information is shown in Table 2.

**Table 2:** Genome sequencing project information

**MIGS ID**	**Property**	**Term**
MIGS 31	Finishing quality	Level 2: High-Quality Draft
MIGS-28	Libraries used	Illumina Std. shotgun library
MIGS 29	Sequencing platforms	Illumina HiSeq 2000
MIGS 31.2	Fold coverage	99.6×
MIGS 30	Assemblers	Velvet v. 1.1.04, ALLPATHS v. R37654
MIGS 32	Gene calling method	Prodigal 2.5
	Locus Tag	C513
	GenBank ID	ARGE00000000
	GenBank Date of Release	September 16, 2013
	GOLD ID	Gp0013605
	BIOPROJECT	174982
MIGS 13	Source Material Identifier	DSM 19884^T^
	Project relevance	Tree of Life, GEBA-KMG

### Growth conditions and genomic DNA preparation

*B. chironomi* strain AIMA4^T^, DSM 19884T, was grown in DSMZ medium 1 (Nutrient Agar), at 28°C [24]. DNA was isolated from 0.5-1 g of cell paste using JetFlex Genomic DNA Purification Kit (GENOMED) following the standard protocol as recommended by the manufacturer, however with additional 50 μl protease K (20 mg/ml) during digest for 60 min. at 58°C. Protein precipitation was done with additional 200 μl Protein Precipitation Buffer, followed by over night incubation on ice. DNA is available through the DNA Bank Network [25].

### Genome sequencing and assembly

The draft genome of *B. chironomi* strain AIMA4^T^ was generated using the Illumina technology [23],[26]. An Illumina standard shotgun library was constructed and sequenced using the Illumina HiSeq 2000 platform which generated 14,014,260 reads totaling 2,102.1 Mb. All general aspects of library construction and sequencing performed at the JGI can be found at the institute website [27]. All raw Illumina sequence data was passed through DUK, a filtering program developed at JGI, which removes known Illumina sequencing and library preparation artifacts [28]. Following steps were then performed for assembly: (1) filtered Illumina reads were assembled using Velvet [29], (2) 1–3 Kbp simulated paired end reads were created from Velvet Contigs using wgsim [30], (3) Illumina reads were assembled with simulated read pairs using Allpaths–LG [31]. Parameters for assembly steps were: (1) Velvet (velveth: 63 –shortPaired and velvetg: −very clean yes –export-Filtered yes –min contig lgth 500 –scaffolding no –cov cutoff 10) (2) wgsim (−e 0 –1 100 –2 100 –r 0 –R 0 –X 0) (3) Allpaths–LG (PrepareAllpathsInputs: PHRED 64 = 1 PLOIDY = 1 FRAG COVERAGE = 125 JUMP COVERAGE = 25 LONG JUMP COV = 50, RunAllpathsLG: THREADS = 8 RUN = std shredpairs TARGETS = standard VAPI WARN ONLY = True OVERWRITE = True). The final draft assembly contained 36 contigs in 36 scaffolds. The total size of the genome is 2.5 Mbp and the final assembly is based on 249.2 Mbp of Illumina data, which provides an average 99.6 × coverage of the genome.

### Genome annotation

Genes were identified using Prodigal [32] as part of the DOE-JGI genome annotation pipeline [33],[34], following by a round of manual curation using the JGI GenePRIMP pipeline [35]. The predicted CDSs were translated and searched against the Integrated Microbial Genomes (IMG) non-redundant database, UniProt, TIGERFam, Pfam, PRIAM, KEGG, COG, and InterPro databases. These data sources were combined to assert a product description for each predicted protein. Additional gene prediction analysis and functional annotation was performed within the Integrated Microbial Genomes-Expert Review (IMG-ER) platform [36].

## Genome properties

The assembly of the draft genome sequence consists of 36 scaffolds amounting to 2,509,395 bp, and the G + C content is 63.5% (Table 3). Of the 2,450 genes predicted, 2,382 were protein-coding genes, and 68 RNAs. The majority of the protein-coding genes (85.5%) were assigned a putative function while the remaining ones were annotated as hypothetical proteins. The distribution of genes into COGs functional categories is presented in Table 4.

**Table 3:** Genome statistics

**Attribute**	**Value**	**% of Total**
Genome size (bp)	2,509,395	100.00%
DNA coding (bp)	2,294,427	91.43%
DNA G + C (bp)	1,593,935	63.52%
DNA scaffolds	36	100.00%
Total genes	2,450	100.00%
Protein coding genes	2,382	97.22%
RNA genes	68	2.78%
Pseudo genes	0	0
Genes in internal clusters	1,788	72.98%
Genes with function prediction	2,095	85.51%
Genes assigned to COGs	1,829	74.65%
Genes with Pfam domains	2,129	86.90%
Genes with signal peptides	171	6.98%
Genes with transmembrane helices	505	20.61%
CRISPR repeats	0	0

**Table 4:** Number of genes associated with the general COG functional categories

**Code**	**Value**	**% age**	**Description**
J	149	7.44	Translation, ribosomal structure and biogenesis
A	1	0.05	RNA processing and modification
K	104	5.19	Transcription
L	106	5.29	Replication, recombination and repair
B	1	0.05	Chromatin structure and dynamics
D	26	1.30	Cell cycle control, cell division, chromosome partitioning
V	32	1.60	Defense mechanisms
T	60	3.00	Signal transduction mechanisms
M	122	6.09	Cell wall/membrane/envelope biogenesis
N	15	0.75	Cell motility
U	60	3.00	Intracellular trafficking, secretion, and vesicular transport
O	95	4.75	Posttranslational modification, protein turnover, chaperones
C	137	6.84	Energy production and conversion
G	66	3.30	Carbohydrate transport and metabolism
E	182	9.09	Amino acid transport and metabolism
F	54	2.70	Nucleotide transport and metabolism
H	113	5.64	Coenzyme transport and metabolism
I	103	5.14	Lipid transport and metabolism
P	115	5.74	Inorganic ion transport and metabolism
Q	52	2.60	Secondary metabolites biosynthesis, transport and catabolism
R	227	11.34	General function prediction only
S	180	8.99	Function unknown
-	621	25.35	Not in COGs

## Insights from the genome sequence

Strain AIMA4^T^ was isolated from chironomid egg masses. Using pyrosequencing method, we have recently shown that the prevalence of *Brachymonas* in the endogenous bacterial communities of chironomid egg masses and larva was 0.04% and 0.006%, respectively [37]. Chironomid tolerance towards pollution is well documented [38]. Senderovich and Halpern [37],[39], demonstrated by using Koch’s postulates that endogenous bacteria in chironomids have a role in protecting the insect from toxicants. Although *B. chironomi* was isolated from chironomid egg masses, its features regarding its protective potential have never been examined. Nevertheless, its genome reveals the potential of this species to protect its host in polluted environments. Genes encoding arsenate detoxification are present in *B. chironomi* strain AIMA4^T^. These genes include an arsenical resistance gene cluster with candidates for transcriptional regulator, ArsR; arsenical resistance operon trans-acting repressor, ArsD; arsenite efflux ATP-binding protein, ArsA and a hypothetical arsenic resistance protein (ACR3 family). A gene for arsenate reductase (ArsC family) is present in a different operon. More genes which may indicate the potential of this bacterium to tolerate or detoxify metals are: copper resistance protein D, CopD; copper chaperone, copper-resistance protein, CopA; copper (or silver) translocating P-type ATPase; uncharacterized lipoprotein NlpE involved in copper resistance; magnesium Mg(2+) and cobalt Co(2+) transport protein, CorA. Moreover, two genes encoding ABC-type transport system involved in resistance to organic solvents, auxiliary and periplasmic components are also present.

The genome of *B. chironomi* strain AIMA4^T^ reveals the potential of the species to produce a polysaccharide capsule. It includes two gene clusters with candidates for capsule polysaccharide export protein, periplasmic protein involved in polysaccharide export, ABC-type polysaccharide/polyol phosphate transport system, ATPase component, ABC-type polysaccharide/polyol phosphate export systems, permease component and predicted glycosyltransferase involved in capsule biosynthesis. Another feature that is found in the genome of *B. chironomi* AIMA4^T^ is its potential to produce a pilus (or pili). The following predicted genes indicate this ability; type IV pilus assembly protein PilB; type IV pilus secretin PilQ; Tfp pilus assembly proteins PilP, PilO and PilV; type IV prepilin peptidase; prepilin-type N-terminal cleavage/methylation domain and pilus retraction ATPase PilT (indicating the ability of twitching motility).

Tolerance of 2.5% NaCl was described for strain AIMA4^T^ by Halpern et al. [2]. The presence of ABC-type proline/glycine betaine transport system in the genome may explain the way this species can tolerate high salt concentrations. In respect to the ampicillin (beta-lactam) antibiotic resistance, the genome encodes one beta-lactamase class B and a negative regulator of beta-lactamase expression. Three genes encoding two component transcriptional regulators (LuxR family), can be found in the genome of strain AIMA4^T^ and demonstrate quorum sensing skills.

## Conclusions

In the current study, we characterized the genome of *B. chironomi* strain AIMA4^T^ that was isolated from a chironomid egg mass [2]. *B. chironomi* belongs to the family *Comamonadaceae* (order *Bukholderiales*; class *Betaproteobacteria*) (Figure 1). Members of this family are known for their ability to cope with harsh environmental condition such as high concentration of toxic metals and other pollutants like aromatic compounds or polymers [e.g. poly(3-hydroxybutyrate-co-3-hydroxyvalerate) [16]. Likewise, the genome of strain AIMA4^T^ reveals the potential of this species to cope with toxic metals. These demonstrate that *B. chironomi* may have a role in protecting its aquatic host (chironomids) in polluted environments.

## Abbreviations

KMG: One thousand microbial genomes

PHBV: Poly(3-hydroxybutyrate-co-3-hydroxyvalerate)

## Competing interest

The authors declare that they have no competing interests.

## Authors’ contributions

MH (Halpern) isolated and characterized strain *B. chironomi* AIMA4^T^; SL, MH (Halpern), HPK and NCK drafted the manuscript. AL, JH, MH (Haynes), TBKR, MH (Huntemann), AP, NNI, KM, VM and TW sequenced, assembled and annotated the genome. EL provided the biomass for DNA extraction and collected data about the organism. MR performed electron microscopy. All authors read and approved the final manuscript.
